# Revisiting where to apply preimplant mapping to improve P‐wave sensing of insertable cardiac monitors

**DOI:** 10.1002/joa3.12739

**Published:** 2022-05-27

**Authors:** Yuhei Kasai, Jungo Kasai, Syuichi Sahashi, Sandeep Shakya, Hiroki Kuji, Naoki Hayakawa, Kotaro Miyaji, Junji Kanda

**Affiliations:** ^1^ Department of Cardiology Kokuho Asahi Chuo Hospital Chiba Japan; ^2^ Paul G. Allen School of Computer Science & Engineering University of Washington Seattle WA USA

**Keywords:** Insertable cardiac monitor, preimplant mapping, P‐wave sensing, reveal LINQ™

## Abstract

**Background:**

Insertable cardiac monitors (ICMs) are used for long‐term cardiac rhythm monitoring. They have proven useful in diagnosing arrhythmias. They are conventionally inserted at the 4th intercostal space without preimplant mapping.

**Method:**

We develop a new method, VisP, that finds an optimal insertion position by applying the lightweight preimplant mapping to nine candidate positions beyond the conventional ones. We retrospectively analyze consecutive 60 patients who underwent ICM insertion (Reveal LINQ™) between April 2019 and March 2021 and compare the two groups with and without VisP.

**Results:**

After 9 patients were excluded because of ectopic atrial rhythms or atrial fibrillation, 51 patients were analyzed. Thirty‐one patients underwent the conventional insertion (non‐mapping), whereas 20 patients underwent VisP. VisP achieved large P‐wave amplitudes while retaining the R‐wave amplitude for all patients; in contrast, P waves were not detected for 11 patients out of the 31 patients in the non‐mapping group (35%). On average, the P‐wave amplitude was 0.065 mV for VisP, compared to 0.029 mV for the non‐mapping group (*p*‐value< .001). The average R‐wave amplitude was 0.69 mV for VisP and 0.71 mV for non‐mapping (*p*‐value = .88), indicating the R‐wave difference is insignificant between the two groups. VisP selected the 4th, 3rd, and 2nd intercostal spaces for 7, 11, and 2 patients, respectively, meaning that 13 out of the 20 cases (65%) fell out of the conventional insertion location of the 4th intercostal space.

**Conclusions:**

VisP improves the diagnostic ability of ICMs by finding an optimal position that yields reliable sensing of P waves while keeping high R‐wave sensing.

## Graphical abstract

We proposed a novel ICM insertion method, Visualize P (VisP), that improves the diagnostic ability of ICMs. We demonstrated that VisP only requires fast, lightweight premapping and finds an optimal position that yields reliable sensing of P waves while keeping high R‐wave sensing.

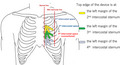

## 1 INTRODUCTION

An implantable cardiac monitor (ICM) is a subcutaneously implanted device that can continuously monitor patients' heart rhythms for up to several years. An ICM is useful to diagnose unexplained syncope and arrhythmias, providing a long‐term opportunity to obtain a symptom‐rhythm correlation.^1^, ^2^, ^3^ Moreover, an ICM has been used to detect occult cardiac arrhythmias such as subclinical atrial fibrillation (AF) after an embolic stroke of an undetermined source (ESUS).^4^, ^5^, ^6^ Reliable R‐wave sensing is particularly important for patients receiving an ICM. Previous studies have shown that an implant location at the 4th intercostal space with a 0–45 degree angle offers sufficiently adequate R‐wave sensing regardless of the patient, and, therefore, preimplant mapping to gain an acceptable R‐wave amplitude is not necessary.^7^, ^8^, ^9^ The R wave is essential to detect an arrhythmia and determine whether it is tachycardia or bradycardia, but information from P waves is also necessary to make a more fine‐grained diagnosis. For instance, tachycardia can be supraventricular tachycardia (SVT) or sinus tachycardia. Bradycardia can be sinus bradycardia, sick sinus syndrome, or an atrioventricular (AV) block. All of these distinctions are key in the treatment of arrhythmias. However, when the ICM of Reveal LINQ™ is used, P waves are clearly visible only 50%–60% of the time.^10^ BIOMONITORIII™, on the contrary, achieves clearer P‐wave sensing at the expense of the much larger device size.^11^ We introduce a new method, called Visualize P (VisP), that improves Reveal LINQ's P‐wave sensing while retaining the accuracy of its R‐wave sensing. Our method challenges the convention that inserts an ICM at the predetermined location (i.e., the 4th intercostal space with a 0–45 degree angle), by applying fast, lightweight preimplant mapping over nine candidate positions to *each individual* patient.

## 2 METHOD

Here, we present our studies to assess the effectiveness of our insertion method, VisP. Our studies involve 60 patients that experienced recurrent syncope or ESUS.

### 2.1 Patients and study design

**FIGURE 1:**
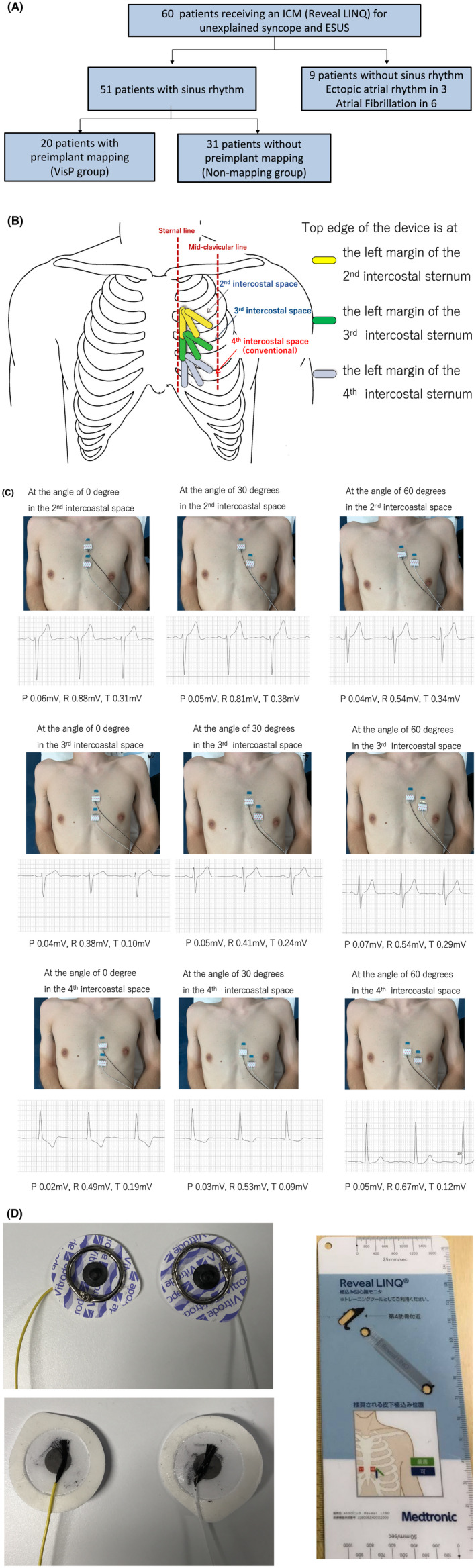
(A) Breakdown of the 60 patients in our studies. Nine patients were excluded because of an ectopic atrial rhythm or atrial fibrillation. (B) Illustration of the nine candidate positions where VisP is applied. The conventional method only considers the 4th intercostal space. (C) VisP premapping applied to the nine positions for a patient. (D) Bipolar electrode with metal markers (left) and the provided marking sheet (right) for reveal LINQ™.

We analyze cases of 60 consecutive patients who received the Reveal LINQ™ (Medtronic, Minneapolis, MN) ICM during the period of April 2019 through March 2021 in our institution (see Figure 1A for the breakdown of the cases). The indication for implantation was recurrent syncope of an unknown origin or ESUS. Nine patients without sinus rhythm (three with an ectopic atrial rhythm and six with atrial fibrillation) were excluded from this study because P waves from the sinus node were not observed. From April 2019 through March 2020, ICM devices were implanted at a conventional position (30‐degree angle at the 4th intercostal space) without preimplant mapping (non‐mapping group, *n* = 31). From April 2020 until March 2021, in contrast, ICM devices were implanted at the optimal position determined by our novel VisP preimplant mapping (VisP group, *n* = 20) (Figure 1A).

In this work, we analyze these past cases to examine the efficacy of our VisP method in achieving adequate sensing of both the R and P waves.

### 2.2 The procedure of VisP preimplant mapping and ICM implantation

VisP applies preimplant mapping more extensively than previously considered: the 2nd and 3rd intercostal spaces as well as the 4th, each with three different angles of 0, 30, and 60 degrees (a total of nine positions). The nine positions are depicted in Figure 1B,C. Preimplant mapping estimates the amplitudes of the P, R, and T waves at each position. The whole premapping procedure takes less than 10 min on average. Based on these estimates, we choose the optimal position out of the nine positions. VisP was performed on 20 patients, and the resulting performance is compared to 31 patients who went through the conventional method previously.

Out of the 9 candidate positions, VisP applies preimplant mapping and chooses the one with the largest estimated P‐wave amplitude that meets the following two conditions:

1. Estimated R‐wave amplitude >0.3 mV.
2. Estimated R‐to‐T wave amplitude ratio >1.7 and T‐wave amplitude <0.5 mV.

The reason why we had Condition (1) is because an R‐amplitude of at least 0.2 mV is required for arrhythmia detection on Reveal LINQ™ (see the official device specifications).^12^ The margin of 0.1 mV (0.3 vs. 0.2 mV) is intended to take the estimation error into consideration. Condition (2) prevents T‐wave *oversensing* in automatic R‐wave sensing. In particular, in order to ensure that the R wave is detected without T‐wave oversensing, Reveal LINQ™ requires that the R‐to‐T ratio in wave amplitude should be larger than 1.54 (i.e., 1/0.65) and the T‐wave amplitude should be smaller than 0.65 mV (see the official clinician manual for more detail).^13^ The margins (R‐to‐T 1.7 vs. 1.54; 0.5 vs. 0.65 mV) are again taken for estimation errors. All 20 patients had at least one position out of the nine candidates that meets both two conditions.

To conduct preimplant mapping, we place two small metal markers that indicate the position in the fluoroscopic image (Figure 1D). A bipolar electrode is used to manually measure the amplitudes of the P, R, and T waves at the angles of 0, 30, and 60 degrees in the 2nd, 3rd, and 4th intercostal spaces respectively by averaging over three beats with band‐pass filters of 20–100 Hz (nine positions in total). The electrode size and spacing were 5 mm and 37.7 mm. We analyzed the results with an RMC‐5000 EP system.

Two experienced electrophysiologists performed the conventional and VisP procedures, while one of them (Operator 1) performed approximately 90% of the cases both with the conventional and VisP methods (Table 1). All ICMs were implanted at the same electrophysiology lab under local anesthesia. In the group with the conventional method (i.e., non‐mapping group), ICM devices were implanted near the left parasternal area over the 4th intercostal space at a 30‐degree angle to the sternum without preimplant mapping. In the VisP group, on the contrary, we chose the optimal position by the VisP method discussed above. We implanted ICMs using the standard tool provided with Reveal LINQ™ (Figure 1D, right).

**TABLE 1:** Patient characteristics. No significant differences between the two groups

Background	VisP group*n* = 20	Non‐mapping group*n* = 31	*p*‐value
Age	71.8 ± 8.6	68.9 ± 12.5	.381
Male	14 (70%)	19 (61%)	.565
Height (cm)	161.5 ± 9.7	160.4 ± 10.2	.713
Body weight (kg)	62.5 ± 11.7	59.6 ± 10.5	.348
BMI (kg/m^2^)	24.0 ± 3.4	23.1 ± 3.0	.338
Indication (Syncope/ESUS)	(8/12)	(15/16)	.580
Operator 1	18 (90%)	28 (90%)	.808
History of atrial arrhythmia	3 (15%)	4 (13%)	.721
Left atrium diameter (mm)	38.5 ± 9.7	36.8 ± 8.5	.692

### 2.3 Study endpoints

The primary endpoint was to compare the amplitudes of the P, R, and T waves between the two groups of non‐mapping and VisP after insertion (Section 3.3). We aim to achieve a sufficiently large P‐wave amplitude while retaining a sufficiently large R‐wave amplitude without T‐wave oversensing. We set the following three criteria to evaluate the success after insertion: (1) the P‐wave amplitude is greater than 0.03 mV; (2) the R‐wave amplitude is greater than 0.2 mV; (3) the R‐to‐T ratio is greater than 1.5. These criteria are all based on the official specifications of Reveal LINQ™.^13^ The secondary endpoint was to assess the accuracy of preimplant mapping estimation by analyzing the resulting amplitudes of the P, R, and T waves after the insertion at the optimal position chosen by VisP (Section 3.4).

### 2.4 Statistical analysis

All values are expressed as median ± standard deviation. We used the unpaired Student's *t*‐test to compare the values between the two groups. Categorical variables were analyzed by Fisher's exact test. A *p*‐value of <.05 indicates statistical significance. The statistical package of JMP® 16.0 (SAS Institute Inc, Cary, NC, USA) was used for all analysis.

## 3 RESULTS

### 3.1 Study population

Out of the 60 patients who underwent Reveal LINQ™ (Medtronic, Minneapolis, MN) device implantation from April 2019 to March 2021, 9 patients were excluded, leading to 51 patients in this study (33 men, average age of about 70 in both groups). Indications for implantation were syncope (45%) and ESUS (55%). As shown in Table 1, we did not find any statistically significant difference between the two groups regarding the patient characteristics (e.g., age, gender, weight). All patients tolerated the procedure without any complications.

### 3.2 VisP procedure

**FIGURE 2:**
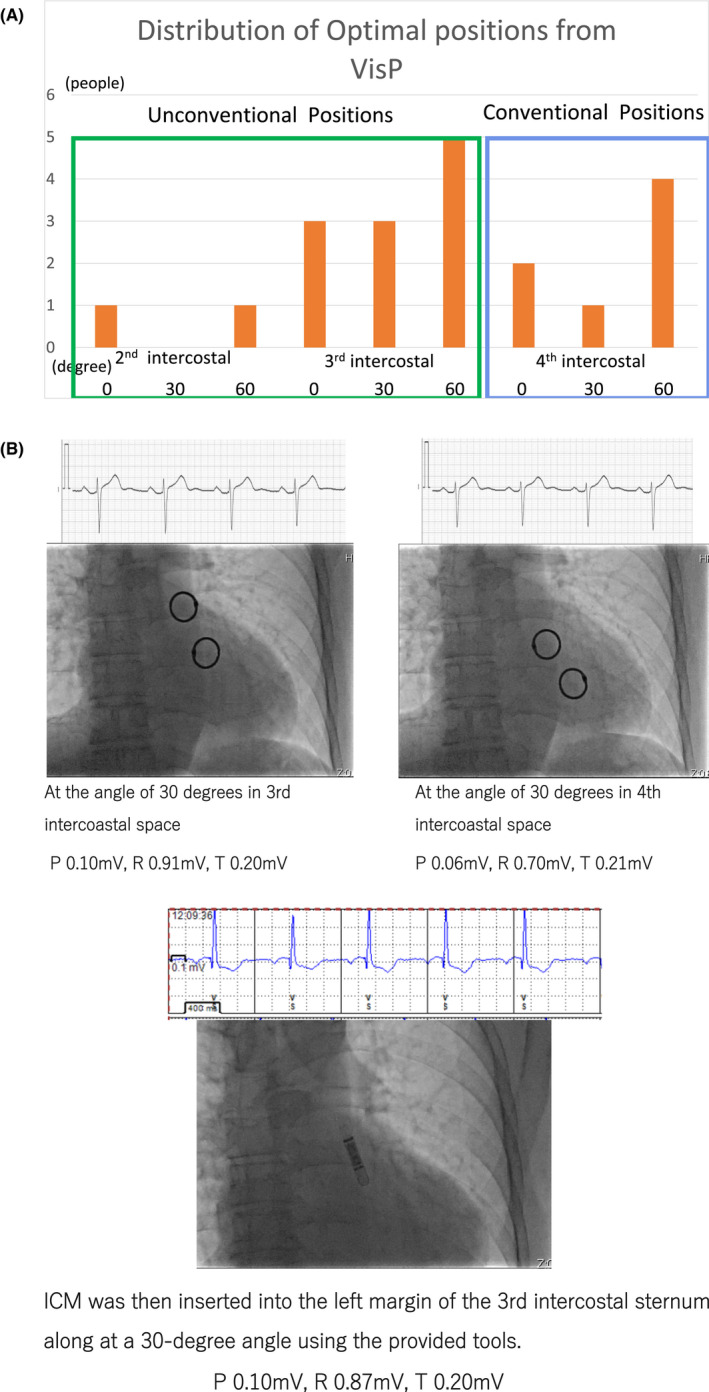
(A) Distribution of the chosen insertion positions for the 20 patients in the VisP group. 65% fell out of the conventional 4th intercostal space. (B) Example premapping results for a patient on the 3rd and 4th intercostal spaces with 30 degrees. They both meet our VisP conditions, but the 3rd one (left) is selected because the P‐wave amplitude is estimated to be higher (0.10 mV vs. 0.06 mV). The bottom picture shows results after insertion.

Figure 2A shows the distribution of the chosen insertion positions for the 20 patients in the VisP group. We see that 13 out of the 20 cases (65%) fell out of the conventional location of the 4th intercostal space: 11 in the 3rd and 2 in the 2nd. Table 2A–C compares the 13 (unconventional) and 7 (conventional) cases in terms of the 12‐lead ECG characteristics. This indicates the importance of exploring all nine positions through the VisP preimplant mapping. We note that the whole VisP procedure took 9 min 32 s on average, implying that VisP only incurs minimal burden on the patients and practitioners. Figure 2B illustrates a case from the VisP group. Here, those two positions (30 degrees in the 3rd and 4th intercostal spaces) meet the two conditions (Section 2.2), but since the estimated P‐wave amplitude was larger in the former, we chose the former, the 3rd intercostal space. Table 2D reports the percentages of conditions met over 9 positions from the 20 patients.

**TABLE 2:** (A) 12‐lead ECG results that compare the 13 cases with implantations in the unconventional positions (2nd or 3rd intercostal) and the 7 cases in the conventional position (4th intercostal); (B) 12‐lead ECG results that compare the 13 cases with implantations in the unconventional positions (2nd or 3rd intercostal) and the 7 cases in the conventional position (4th intercostal); (C) 12‐lead ECG results that compare the 13 cases with implantations in the unconventional positions (2nd or 3rd intercostal) and the 7 cases in the conventional position (4th intercostal); (D) The numbers and percentages of the patients that meet each condition or both for every mapping location

(A)			
P wave from 12‐lead ECG	Unconventional position	Conventional position	*p*‐value
I lead (mV)	0.048 ± 0.025	0.063 ± 0.013	.152
II lead (mV)	0.065 ± 0.026	0.119 ± 0.013	*.048*
III lead (mV)	0.058 ± 0.029	0.076 ± 0.041	.267
aVR lead (mV)	0.057 ± 0.025	0.079 ± 0.034	.124
aVL lead (mV)	0.042 ± 0.025	0.033 ± 0.015	.403
aVF lead (mV)	0.057 ± 0.010	0.098 ± 0.013	*.025*

(B)			
R wave from 12‐lead ECG	Unconventional position	Conventional position	*p*‐value
I lead (mV)	0.725 ± 0.295	0.630 ± 0.013	.447
II lead (mV)	0.853 ± 0.711	0.694 ± 0.302	.577
III lead (mV)	0.752 ± 0.494	0.397 ± 0.194	.087
aVR lead (mV)	0.758 ± 0.404	0.662 ± 0.202	.567
aVL lead (mV)	0.602 ± 0.211	0.423 ± 0.169	.070
aVF lead (mV)	0.676 ± 0.149	0.522 ± 0.202	.548

(C)			
T wave from 12‐lead ECG	Unconventional position	Conventional position	*p*‐value
I lead (mV)	0.116 ± 0.072	0.150 ± 0.074	.331
II lead (mV)	0.182 ± 0.111	0.222 ± 0.103	.429
III lead (mV)	0.116 ± 0.092	0.093 ± 0.045	.539
aVR lead (mV)	0.146 ± 0.084	0.179 ± 0.093	.438
aVL lead (mV)	0.089 ± 0.075	0.056 ± 0.026	.282
aVF lead (mV)	0.142 ± 0.097	0.153 ± 0.067	.801

(D)									
Intercostal Degree	2nd 0°	2nd 30°	2nd 60°	3rd 0°	3rd 30°	3rd 60°	4th 0°	4th 30°	4th 60°
Condition (1)	19 (95%)	19 (95%)	19 (95%)	20 (100%)	20 (100%)	20 (100%)	20 (100%)	20 (100%)	20 (100%)
Condition (2)	17 (85%)	18 (90%)	16 (80%)	18 (90%)	18 (90%)	17 (85%)	17 (85%)	17 (85%)	17 (85%)
Both	16 (80%)	17 (85%)	15 (75%)	18 (90%)	18 (90%)	17 (85%)	17 (85%)	17 (85%)	17 (85%)

Statistical significance (*p*‐value < .05) is indicated in italics.

### 3.3 Primary endpoint: Wave amplitudes and visibility

**FIGURE 3:**
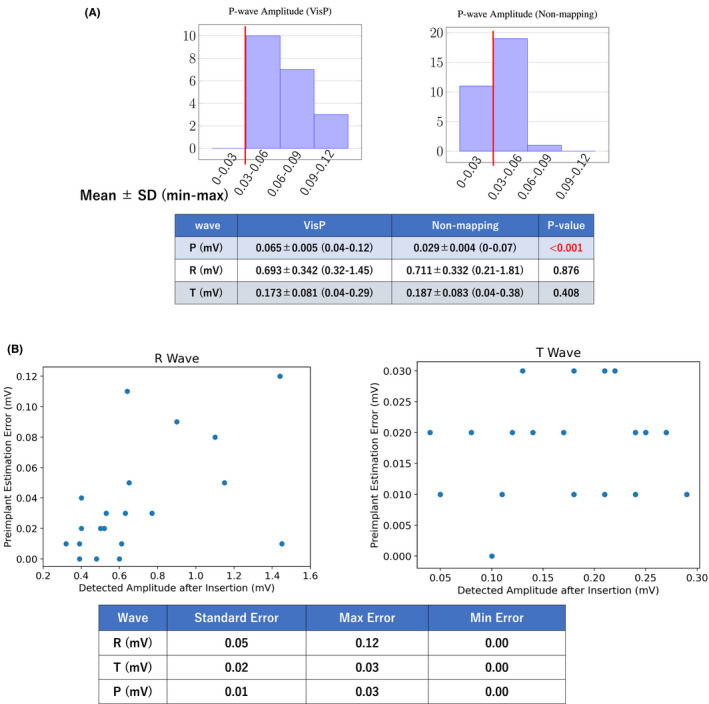
(A) Histograms of the detected P‐wave amplitudes for the VisP and non‐mapping groups after insertion. An amplitude of 0.03 mV is necessary for reliable sensing (indicated by the red vertical lines). The summary statistics for the P, R, T waves are provided. The difference in the P wave is statistically significant, while the other two are not. (B) Analysis of VisP premapping estimation errors for the R and T waves. The plots visualize the relationship between the magnitude of the error and the actual wave amplitude after insertion. Standard, maximum, and minimum errors are also computed.

**FIGURE 4:**
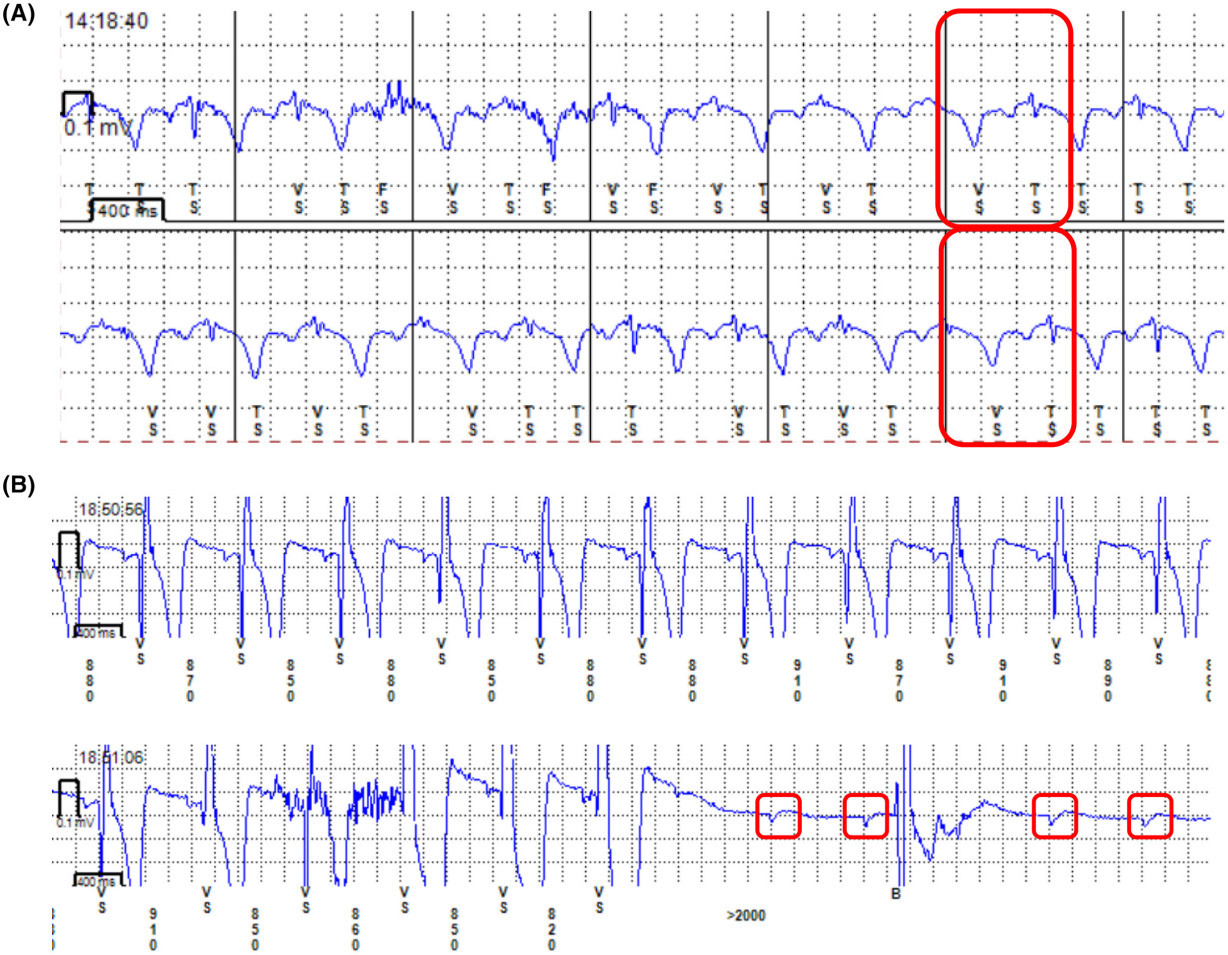
(A) An example patient from the non‐mapping group that had the T‐wave oversensing issue (indicated in the red squares). (B) An example case where our VisP‐based ICM insertion helped us diagnose atrioventricular block, as opposed to the sick sinus syndrome. The red square indicates detected P waves.

Figure 3A compares the two groups in terms of their detected wave amplitudes. Histograms for the detected P‐wave amplitudes and summary statistics are provided for the two groups. We observe that the P‐wave amplitude was sufficiently large (0.04+ mV) for all 20 patients in the VisP group. In contrast, the P‐wave amplitude was more than 0.03 mV only for 20 out of the 31 patients in the non‐mapping group (65%), in line with the previous studies that report 50%–60%.^9^ The difference in R‐wave amplitude was statistically insignificant despite the fact that the insertion was performed on the 2nd or 3rd intercostal space for 65% of the patients in the VisP group. This challenges the commonly held assumption that the 4th intercostal space is most suitable for ICM insertion; preimplant mapping is indeed useful to find a better‐suited position. Note that as seen in Table 3A–C, the 12‐lead ECG before insertion showed no significant difference in the P, R, and T‐wave amplitudes between the two groups. This means that patient‐specific wave amplitudes do not explain the differences that we found between the two groups. Note also that premapping for 3 out of the 20 patients in the VisP group showed the R‐to‐T ratio less than 1.7 in the 4th intercostal space (Condition 2 in Table 2D), and VisP helped us find a better position without T‐wave oversensing. In these cases, the conventional method could have resulted in T‐wave oversensing. In fact, we found two patients who went through the conventional method had the T‐wave oversensing issue (Figure 4A). This suggests that while the primary benefit of VisP is the improvement of P‐wave sensing, it has an additional advantage over the conventional method with respect to reliable R‐wave sensing.

**TABLE 3:** (A) P‐wave amplitudes from the 12‐lead ECG before insertion. No significant differences are found in all limb leads; (B) R‐wave amplitudes from the 12‐lead ECG before insertion. No significant differences are found in all limb leads; (C) T‐wave amplitudes from the 12‐lead ECG before insertion. No significant differences are found in all limb leads.

(A)			
P wave from 12‐lead ECG	VisP group	Non‐mapping group	*p*‐value
I lead (mV)	0.053 ± 0.022	0.043 ± 0.021	.154
II lead (mV)	0.084 ± 0.043	0.086 ± 0.037	0.813
III lead (mV)	0.064 ± 0.034	0.067 ± 0.030	.762
aVR lead (mV)	0.065 ± 0.029	0.059 ± 0.021	.400
aVL lead (mV)	0.039 ± 0.022	0.036 ± 0.016	.587
aVF lead (mV)	0.071 ± 0.039	0.073 ± 0.033	.822

(B)			
R wave from 12‐lead ECG	VisP group	Non‐mapping group	*p*‐value
I lead (mV)	0.692 ± 0.257	0.746 ± 0.281	.484
II lead (mV)	0.797 ± 0.595	0.944 ± 0.358	.274
III lead (mV)	0.628 ± 0.443	0.716 ± 0.320	.416
aVR lead (mV)	0.725 ± 0.343	0.745 ± 0.240	.803
aVL lead (mV)	0.540 ± 0.213	0.540 ± 0.260	.998
aVF lead (mV)	0.623 ± 0.526	0.727 ± 0.356	.401

(C)			
T wave from 12‐lead ECG	VisP group	Non‐mapping group	*p*‐value
I lead (mV)	0.128 ± 0.073	0.148 ± 0.077	.351
II lead (mV)	0.196 ± 0.108	0.213 ± 0.110	.592
III lead (mV)	0.108 ± 0.078	0.114 ± 0.064	.783
aVR lead (mV)	0.158 ± 0.086	0.196 ± 0.089	.136
aVL lead (mV)	0.077 ± 0.063	0.072 ± 0.043	.718
aVF lead (mV)	0.146 ± 0.086	0.152 ± 0.086	.801

### 3.4 Secondary endpoint: Accuracy of the VisP estimates

Figure 3B plots the estimation error (y axis) and the detected wave amplitude (x axis) for the R and T waves. We also compute the standard, maximum, and minimum errors of the estimates. In all cases, the margins that we had in our VisP conditions (Section 2.2) were sufficient to ensure the large R wave amplitude and the small T wave amplitude. Notice that there are outliers where we see relatively large estimation errors for the R wave, but most of them are far away from the threshold (0.2 mV for the R wave), meaning that those errors do not result in selecting an unsuitable position. For all three waves, we also see a high correlation between the estimates and detected amplitudes (P: 0.87; R: 0.98; T: 0.97) again suggesting the effectiveness of the VisP premapping.

## 4 DISCUSSION

Prior studies focused mainly on the R‐wave amplitude and concluded that the 4th intercostal space is the best location regardless of the patient and thus preimplant mapping is unnecessary.^7^, ^8^, ^9^ Our primary study endpoint suggested, however, the 4th intercostal space is suboptimal if P‐wave sensing is also considered. P‐wave sensing from our VisP method allows for a fine‐grained diagnosis of the type of arrhythmias.

In addition to the benefit of improving P‐wave sensing, Condition (2) in VisP avoids T‐wave oversensing.

Seen in Figure 4B are intracardiac electrograms from a case where our VisP‐based ICM insertion indeed helped us diagnose atrioventricular block, as opposed to the sick sinus syndrome. This particular case illustrates the effectiveness of P‐wave detection from VisP‐based ICM insertion. We further analyzed the 31 cases from the non‐mapping group to understand when VisP is particularly beneficial. Specifically, we compared the 12‐lead ECG between the 20 cases where P waves were successfully detected and the rest of the unsuccessful 11 cases (Table 4). The inferior leads (II, III, and aVF) show that the P‐wave amplitude is larger in the 20 patients (statistically significant for III and aVF). This finding suggests that the VisP premapping is especially crucial for patients with small P‐wave amplitudes in the inferior leads.

**TABLE 4:** 12‐lead ECG results that compare the 20 cases with successful P‐wave detection and the 11 unsuccessful cases in the non‐mapping group

P wave from 12‐lead ECG	Successful	Unsuccessful	*p*‐value
I lead (mV)	0.043 ± 0.021	0.048 ± 0.017	.498
II lead (mV)	0.095 ± 0.039	0.071 ± 0.028	.083
III lead (mV)	0.075 ± 0.028	0.051 ± 0.029	*.034*
aVR lead (mV)	0.061 ± 0.024	0.055 ± 0.015	.485
aVL lead (mV)	0.033 ± 0.016	0.042 ± 0.015	.102
aVF lead (mV)	0.083 ± 0.032	0.056 ± 0.029	*.025*

Statistical significance (*p*‐value < .05) is indicated in italics.

Throughout our studies, we assumed the use of Reveal LINQ™, which uses enhanced detection algorithms for high sensitivity and specificity.^14^ There are, however, alternative ICMs. In particular, BIOMONITORIII™ is known to be able to reliably detect P waves as well as R waves. Nonetheless, Reveal LINQ™ is still a better choice for patients with relatively short height (such as the ones considered in our study) because of its compact size. We thus believe that VisP has a large impact on many patients that need ICM insertion.

## 5 LIMITATIONS

This was a retrospective, single‐center, observational study. We analyzed relatively small sets of patients. A prospective study with many patients across varying institutions and countries would be ideal. We also based our assessment on the detected wave amplitudes, but further analyses over a long period of time would be useful. We also note that all preimplant mapping was applied in the spine position to minimize the additional burden on patients and clinicians. Studies using other positions would strengthen our studies in the future.

## 6 CONCLUSIONS

Conventionally, clinical practitioners perform ICM insertion at the 4th intercostal space. We challenged this convention to improve P‐wave sensing while retaining reliable R‐wave sensing. We proposed a novel method, VisP, that finds an optimal insertion position by applying fast, lightweight preimplant mapping to nine candidate positions. Our studies demonstrated that VisP achieves reliable sensing of both the P and R waves simultaneously for all patients. The ICM is a diagnostic yet invasive tool that adds a non‐negligible physical burden on patients. Clinical practitioners should thus make the best effort to obtain as much reliable information as possible. We hope that VisP will enhance the diagnostic ability of the ICM and benefit patients who need its insertion.

## DISCLOSURE

The enrolled patient provided written informed consent. The examination was made in accordance with the approved principles. All the preparations and the equipment used are officially certified for the clinical use.

The protocol for this research project has been approved by a suitably constituted Ethics Committee of the institution and it conforms to the provisions of the Declaration of Helsinki. Committee of Kokuho Asahi Chuo Hospital, Approval No. 2020012107 (Approval date 9 December 2020).

## CONFLICT OF INTEREST

The authors declare no conflict of interest for this article.
